# Cardiovascular magnetic resonance in light-chain amyloidosis to guide treatment

**DOI:** 10.1093/eurheartj/ehac363

**Published:** 2022-07-26

**Authors:** Ana Martinez-Naharro, Rishi Patel, Tushar Kotecha, Nina Karia, Adam Ioannou, Aviva Petrie, Liza A Chacko, Yousuf Razvi, Sriram Ravichandran, James Brown, Steven Law, Cristina Quarta, Shameem Mahmood, Brendan Wisniowski, Silvia Pica, Sajitha Sachchithanantham, Helen J Lachmann, James C Moon, Daniel S Knight, Carol Whelan, Lucia Venneri, Hui Xue, Peter Kellman, Julian D Gillmore, Philip N Hawkins, Ashutosh D Wechalekar, Marianna Fontana

**Affiliations:** National Amyloidosis Centre, Division of Medicine, University College London, Royal Free Hospital, London, UK; National Amyloidosis Centre, Division of Medicine, University College London, Royal Free Hospital, London, UK; National Amyloidosis Centre, Division of Medicine, University College London, Royal Free Hospital, London, UK; Institute of Cardiovascular Science, University College London, London, UK; National Amyloidosis Centre, Division of Medicine, University College London, Royal Free Hospital, London, UK; Institute of Cardiovascular Science, University College London, London, UK; National Amyloidosis Centre, Division of Medicine, University College London, Royal Free Hospital, London, UK; UCL Eastman Dental Institute, London, UK; National Amyloidosis Centre, Division of Medicine, University College London, Royal Free Hospital, London, UK; National Amyloidosis Centre, Division of Medicine, University College London, Royal Free Hospital, London, UK; National Amyloidosis Centre, Division of Medicine, University College London, Royal Free Hospital, London, UK; National Amyloidosis Centre, Division of Medicine, University College London, Royal Free Hospital, London, UK; Institute of Cardiovascular Science, University College London, London, UK; National Amyloidosis Centre, Division of Medicine, University College London, Royal Free Hospital, London, UK; National Amyloidosis Centre, Division of Medicine, University College London, Royal Free Hospital, London, UK; National Amyloidosis Centre, Division of Medicine, University College London, Royal Free Hospital, London, UK; National Amyloidosis Centre, Division of Medicine, University College London, Royal Free Hospital, London, UK; National Amyloidosis Centre, Division of Medicine, University College London, Royal Free Hospital, London, UK; National Amyloidosis Centre, Division of Medicine, University College London, Royal Free Hospital, London, UK; National Amyloidosis Centre, Division of Medicine, University College London, Royal Free Hospital, London, UK; Institute of Cardiovascular Science, University College London, London, UK; Barts Heart Centre, West Smithfield, London, UK; National Amyloidosis Centre, Division of Medicine, University College London, Royal Free Hospital, London, UK; Institute of Cardiovascular Science, University College London, London, UK; National Amyloidosis Centre, Division of Medicine, University College London, Royal Free Hospital, London, UK; National Amyloidosis Centre, Division of Medicine, University College London, Royal Free Hospital, London, UK; National Heart, Lung and Blood Institute, National Institutes of Health, Bethesda, MD, USA; National Heart, Lung and Blood Institute, National Institutes of Health, Bethesda, MD, USA; National Amyloidosis Centre, Division of Medicine, University College London, Royal Free Hospital, London, UK; National Amyloidosis Centre, Division of Medicine, University College London, Royal Free Hospital, London, UK; National Amyloidosis Centre, Division of Medicine, University College London, Royal Free Hospital, London, UK; National Amyloidosis Centre, Division of Medicine, University College London, Royal Free Hospital, London, UK

**Keywords:** CMR, Amyloidosis, T1 mapping, ECV

## Abstract

**Aims:**

To assess the ability of cardiovascular magnetic resonance (CMR) to (i) measure changes in response to chemotherapy; (ii) assess the correlation between haematological response and changes in extracellular volume (ECV); and (iii) assess the association between changes in ECV and prognosis over and above existing predictors.

**Methods and results:**

In total, 176 patients with cardiac AL amyloidosis were assessed using serial N-terminal pro-B-type natriuretic peptide (NT-proBNP), echocardiography, free light chains and CMR with T1 and ECV mapping at diagnosis and subsequently 6, 12, and 24 months after starting chemotherapy. Haematological response was graded as complete response (CR), very good partial response (VGPR), partial response (PR), or no response (NR). CMR response was graded by changes in ECV as progression (≥0.05 increase), stable (<0.05 change), or regression (≥0.05 decrease). At 6 months, CMR regression was observed in 3% (all CR/VGPR) and CMR progression in 32% (61% in PR/NR; 39% CR/VGPR). After 1 year, 22% had regression (all CR/VGPR), and 22% had progression (63% in PR/NR; 37% CR/VGPR). At 2 years, 38% had regression (all CR/VGPR), and 14% had progression (80% in PR/NR; 20% CR/VGPR). Thirty-six (25%) patients died during follow-up (40 ± 15 months); CMR response at 6 months predicted death (progression hazard ratio 3.82; 95% confidence interval 1.95–7.49; *P* < 0.001) and remained prognostic after adjusting for haematological response, NT-proBNP and longitudinal strain (*P* < 0.01).

**Conclusions:**

Cardiac amyloid deposits frequently regress following chemotherapy, but only in patients who achieve CR or VGPR. Changes in ECV predict outcome after adjusting for known predictors.

## Structured Graphical Abstract

**Structured Graphical Abstract:**
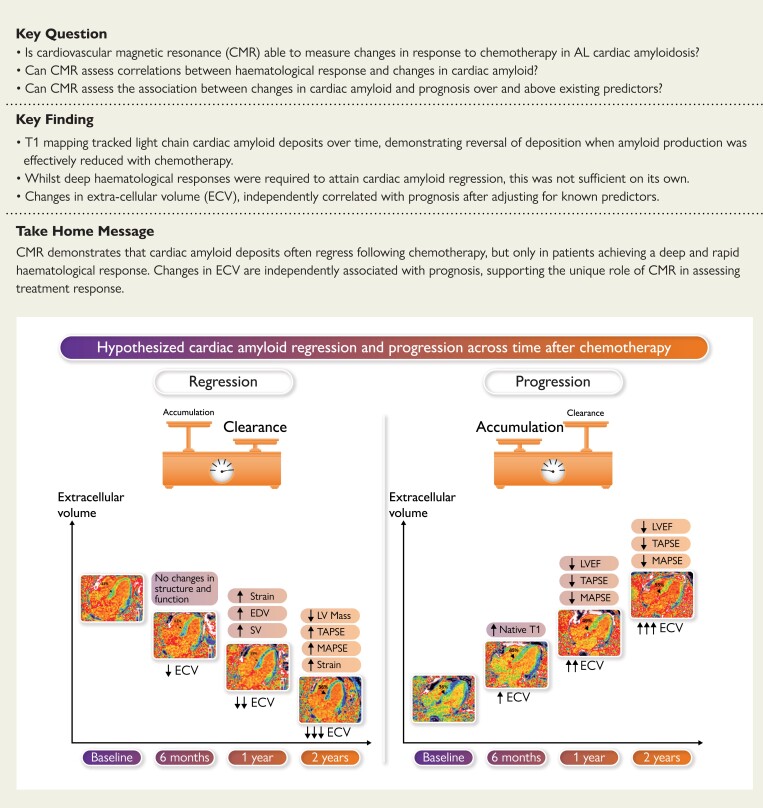
ECV, extracellular volume; EDV, end-diastolic volume; LV, left ventricle; LVEF, LV ejection fraction; MAPSE, mitral annular plane systolic excursion; TAPSE, tricuspid annular plane systolic excursion.

**See the editorial comment for this article ‘The challenge of managing patients with light-chain cardiac amyloidosis: the value of cardiac magnetic resonance as a guide to the treatment response’, by Thibaud Damy *et al*., https://doi.org/10.1093/eurheartj/ehac526.**

## Introduction

Systemic light-chain (AL) amyloidosis is a complication of clonal B-cell disorders, characterized by deposition in the interstitial space of aggregated misfolded monoclonal immunoglobulin light-chains in the form of amyloid fibrils. The presence and severity of cardiac involvement in AL amyloidosis is the main driver of prognosis;^1^ patients with symptomatic heart failure frequently die within 6 months^1^ but median survival has nearly doubled over the past decade, mainly due to the remarkable progress in chemotherapy.

The direct effect of chemotherapy is principally evaluated with serial measurements of serum-free light chains (FLC),^2^ defining haematological response which differs markedly among patients.

Serum concentration of brain natriuretic peptides and echocardiographic parameters are currently the reference standards for assessing cardiac organ responses to chemotherapy.^3–5^ Brain natriuretic peptides have been extensively utilised in cardiac AL amyloidosis, in the assessment of patients’ prognosis and in the stratification of treatment response, with brain natriuretic peptides emerging over the years as having a key role in the management of patients with AL amyloidosis.^6^ Furthermore, there is considerable pre-clinical and clinical evidence that brain natriuretic peptide concentration depends on a direct effect of the circulating amyloid precursor in AL amyloidosis, highlighting the important role of this biomarker in the early phase of active amyloid production.^6^ However, elevation in brain natriuretic peptide levels represent the final common pathway of several mechanisms, including renal impairment,^7^ worsening in fluid status, neurohormonal activation, worsening in cardiac function, and light chain toxicity. Therefore brain natriuretic peptide levels do not accurately represent cardiac amyloid burden.^8^ The typical dearth of significant structural and functional changes on serial echocardiography after successful chemotherapy, other than changes in longitudinal strain in a proportion of patients at one year,^4^ has led to the widespread belief that regression of myocardial amyloid can take place either extremely slowly or not at all.^9^

Cardiovascular magnetic resonance (CMR) with tissue characterization is a sensitive tool for detecting myocardial amyloid deposits. Late gadolinium enhancement (LGE) shows a continuum of cardiac infiltration, from subendocardial LGE to increasing transmurality as the disease progresses.^10^ T1 mapping with extracellular volume (ECV) measurement enables the myocyte and extra-cellular compartments to be measured separately.^11–15^ ECV has been shown to track markers of disease severity,^8^ improve diagnostic accuracy and patient stratification.^11–14,16–20^ We have previously shown, in a small retrospective study with only two time points, that it has the potential to track changes in response to treatment.^21^

The aim of this study was to assess the ability of CMR with ECV mapping to (i) measure changes in response to chemotherapy, (ii) assess the correlation between haematological response and changes in ECV, and (iii) assess the association between changes in ECV and prognosis over and above existing predictors.

## Methods

Study subjects comprised individuals with cardiac involvement identified from a long-term prospective observational study of newly diagnosed AL amyloidosis patients (ALchemy) conducted at the National Amyloidosis Centre (NAC), United Kingdom (January 2016 to December 2018). Patients with no cardiac involvement or patients who had contraindications for CMR (due to renal impairment, pacemaker implantation, or difficulties to lie flat) were not included in this study. Prior to enrolment, the diagnosis of AL amyloidosis was confirmed by central review of histological material inclusive of Congo red staining and cardiac involvement was established by CMR, as per consensus guidelines, by the presence of diffuse subendocardial or transmural LGE, altered gadolinium kinetics and/or diffusely elevated ECV. Amyloid subtype was identified by immunohistochemistry with specific antibodies, or by mass spectrometry. All patients underwent comprehensive assessments including 6-minute walk test (6MWT), electrocardiogram, echocardiography, N-terminal pro-B-type natriuretic peptide (NT-proBNP) measurements and CMR with T1 mapping and ECV measurements at baseline and 6, 12, and 24 months after receiving chemotherapy. FLC measurements and serum immunofixation was performed monthly at the NAC to assess haematological response, which were defined as per international consensus criteria.^22–24^ Briefly, normal FLC levels with normal kappa/lambda ratio and negative serum and urine immunofixation was considered a complete response (CR); a reduction in dFLC (the difference in concentration between the aberrant vs. uninvolved class of FLC) to <40 mg/L denotes a very good partial response (VGPR); >50% reduction in dFLC defines a partial response (PR) whilst no response (NR) comprises less than PR.^22,25^ Cardiac organ response by NT-proBNP is defined by reduction in >30% and >300 ng/L.^24^

### Echocardiography acquisition and analysis

Echocardiographic evaluation was performed using a GE Vivid E9 ultrasound machine equipped with a 5S probe and measurements performed offline using EchoPAC software (Version 202). At least three consecutive beats were recorded for each view, and images were stored for off-line analysis. Left ventricular (LV) chamber morphology was assessed following the latest American Society of Echocardiography/European Association of Cardiovascular Imaging Guideline:^26^ Left atrial area and right atrial area were measured in the four-chamber view. LV ejection fraction (LVEF) was calculated with the biplane Simpson’s method from volumes acquired in both the four-chamber and the two-chamber views. Lateral mitral annular plane systolic excursion (MAPSE) and tricuspid annular plane systolic excursion (TAPSE) were assessed with M-mode in the four-chamber view. LV early (E wave), late (A wave) diastolic filling, its ratio (E/A) were evaluated with pulsed Doppler in the four-chamber view. Lateral and septal mitral annulus velocities (e′ wave) were assessed with tissue Doppler in the four-chamber view; the ratio between the LV early diastolic filling wave and lateral mitral annulus velocity (E/e′) was calculated.^27^ Digitally acquired clips were considered suitable for offline 2D speckle strain imaging analysis if at least three cardiac cycles were available, with high frame rates (70 to 100 frame/s) and without dropout of more than one LV segment or significant foreshortening of the ventricle. The endocardial border was traced at the end-diastolic frame in the apical view. End-diastole was defined by the QRS complex or by the frame just before mitral valve closure. The software tracked speckles along the endocardial and epicardial borders throughout the cardiac cycle, and the width of the region of interest was adjusted to fit the entire myocardium. All strain and strain-derived variables were measured in the apical four-chamber view. Peak longitudinal strain was computed automatically, generating regional data from six segments (basal, mid, apical interventricular septum and basal, mid, apical lateral wall), to calculate an average value. Valvular assessment was performed using an integrated approach as per current guidelines. A clinically meaningful longitudinal strain improvement of −2.0% after initiation of chemotherapy was used to define strain changes (improvement or not improvement), as previously described.^4^

All the echocardiogram analysis was performed blinded to CMR results.

### CMR image acquisition and analysis

All subjects underwent CMR on a 1.5-T clinical scanner (Magnetom Aera, Siemens Healthcare, Erlangen, Germany). Within a conventional clinical scan [localizers and cine imaging with steady state free precession (SSFP) sequence], LGE imaging was acquired with both magnitude inversion recovery and phase-sensitive inversion recovery (PSIR) sequence reconstructions with SSFP read-outs. T1 measurement was performed with the use of the modified look-locker inversion recovery sequence. For T1 mapping, three short axis maps (base, middle and apex) were manually contoured at the endocardial and epicardial border, segmented into an American Heart Association 16-segment model using the right ventricular insertion points. After a bolus of gadoterate meglumine (0.1 mmol/kg, gadolinium-DOTA, Dotarem, Guerbet S.A. France) and LGE imaging, T1 mapping was repeated 15 min post-contrast using the same slice locations with the modified look-locker inversion recovery sequence, to produce automated inline ECV mapping reconstruction. T1-mapping protocols used 5 s(3 s)3s and 4 s(1 s)3 s(1 s)2 s sampling, pre- and post-contrast, respectively.^28^

All CMR image analysis was performed blinded to all other clinical and imaging data. The LGE pattern was classified into three groups according to PSIR LGE transmurality: group 1, no LGE; group 2, subendocardial LGE only; and group 3, transmural LGE. Regression in the cardiac amyloid burden was considered as an absolute increase or decrease of 5% of ECV. This cut-off was selected, as previously described,^21^ based on repeatability exercise data previously published.^29^ The limits of agreement in a Bland-Altman analysis (equal to the mean of repeated measurements in a patient ± 2 times the standard deviation of the differences [SD_d_]) would be expected to encompass 95% of the differences, with 5% of them outside these limits. As 2SD_d_ represents the maximum likely difference in a patient who repeated the analysis, anything greater than this for a measurement taken at different times is likely to be indicative of a real change. This change in ECV was graded as progression (≥5% increase), stable (<5% change) or regression (≥5% decrease). Image analysis was performed offline using Osirix MD 9.0 (Bernex, Switzerland).

### Statistical analysis

Statistical analysis was performed using IBM SPSS Statistics Version 26 (IBM, Somers, New York) for all analyses apart from survival when Stata (StataCorp. 2021. Stata Statistical Software: Release 17. College Station, TX: StataCorp LLC.) was used. All continuous variables were normally distributed (Shapiro-Wilk), other than NT-proBNP, which was natural log-transformed for parametric testing. These are summarized in *Table 1* as mean (standard deviation), apart from the non-transformed NT-proBNP which is presented in *Table 1* as median and interquartile range (IQR). Comparisons between different time points within the same group were performed by paired *t*-test and comparisons between different groups were performed by one-way analysis of variance followed by, if significant, post hoc Bonferroni corrected pairwise comparisons. The assumptions underlying these tests were investigated and confirmed. The chi-square test or Fisher exact test, as appropriate, was used to compare categorical data.

**Table 1:** Baseline characteristics, biomarkers, 6-minute walk test, echocardiographic and cardiovascular magnetic resonance parameters for patients who had amyloid regression, stable findings or progression by cardiovascular magnetic resonance at 6 months

Characteristics	Regression (*N* = 4)	Stable (*N* = 92)	Progression (*N* = 46)
Men, *n* (%)	2 (50%)	58 (63%)	26 (57%)
Age (years)	45 (16)	65 (10)*	63 (11)**
NT-proBNP (pmol/L)	250 (146–296)	2418 (858–5184)*	3730 (883–6544)**
6MWT (m)	476 (153)	409 (135)	401 (131)
**Echocardiographic parameters**			
IVS (cm)	1.07 (SD 0.06)	1.45 (SD 0.24)*	1.44 (SD 0.25)**
LVEDD (cm)	3.77 (SD 0.21)	4.15 (SD 0.56)	4.12 (SD 0.54)
LAA (cm^2^)	14.23 (4.80)	21.45 (SD 5.64)	20.46 (SD 4.72)
E/E’	9 (SD 5)	16 (SD 7)	16 (SD 7)
2D LS	−20.8 (SD 4.5)	−13.8 (SD 4.8)*	-14.1 (SD 5.3)
**CMR parameters**			
LVEDVi (mL/m^2^)	59 (SD 18)	69 (SD 15)	63 (SD 11)
LVESVi (mL/m^2^)	17 (SD 7)	25 (SD 11)	22 (SD 8)
Maximal IVS (mm)	11 (SD 1)	16 (SD 4)	16 (SD 4)
LV mass index (g/m^2^)	60 (SD 19)	99 (SD 32)*	98 (SD 29)
LVSVi (mL/m^2^)	43 (SD 11)	44 (SD 10)	41 (SD 9)
LVEF (%)	73 (SD 4)	64 (SD 9)	65 (SD 10)
LAA (cm^2^)	18 (SD 4)	28 (SD 8)*	27 (SD 5)**
RA area (cm^2^)	16 (SD 3)	23 (SD 7)	24 (SD 6)
MAPSE (mm)	11 (SD 3)	8 (SD 3)	9 (SD 3)
TAPSE (mm)	21 (SD 4)	16 (SD 5)	16 (SD 6)
Native T1 (ms)	1157 (SD 50)	1151 (SD 64)	1152 (SD 51)
T2 (ms)	54 (SD 6)	53 (SD 3)	53 (SD 3)
ECV (%)	41 (SD 4)	47 (SD 7)	45 (SD 7)

6MWT, 6-minute walk test; AL, systemic light-chain; CMR, cardiovascular magnetic resonance; CR: complete (haematological) response, DT, deceleration time; ECV, extracellular volume; LS, longitudinal strain; IVS, interventricular septum; LAA, left atrial area; LPW, left posterior wall; LV, left ventricle; LVEDD, left ventricular end-diastolic diameter; LVEDV/LVEDVi, left ventricular end-diastolic volume/index; LVEF, left ventricular ejection fraction; LVESV/LVESVi, left ventricular end-systolic volume/indexed; LVSV/LVSVi, left ventricular stroke volume/index; LVEF, left ventricular ejection fraction; NR, no (haematological) response; NT-proBNP, N-terminal pro-B-type natriuretic peptide; PR, partial (haematological) response; TAPSE, tricuspid annular plane systolic excursion; VGPR, very good partial (haematological) response.

All continuous variables are presented as mean and standard deviation apart from NT-proBNP which is presented as median and interquartile range. Natural log-transformed NT-proBNP was used for parametric testing, but in this table the raw data is summarized by the median and interquartile range.

*P*-values for pairwise comparison:

* *P* < 0.05 for regression vs. stable

** *P* < 0.05 for regression vs. progression.

Survival was evaluated by Cox proportional hazards regression analysis, which provided estimated hazard ratios (HRs) with 95% confidence intervals (CIs) and Kaplan–Meier curves. The proportional hazards assumption in each analysis was investigated and confirmed.

Variability of ECV measurements was assessed calculating intraclass correlation coefficients (ICC) for the interobserver and intraobserver variation obtaining excellent correlation with an ICC for the interobserver variation of 0.997 and an ICC for the intraobserver variation of 0.998. Precision of the technique was previously published.^29^

A multivariable model was used to investigate factors associated with overall survival. All variables were selected a priori for clinical relevance: change in ECV as a marker of amyloid infiltration; change in NT-proBNP as a blood biomarker, change in longitudinal strain as an echocardiographic variable and haematological response. The small group of better ECV at 6 months (4 patients) was not considered for the survival analysis at 6 months. Collinearity among the variables used for the multivariate model was excluded, obtaining a mean variance inflation factor of 1.17. Also, there was no evidence that the proportional hazards assumption was violated.

All *P*-values were two-sided with a significance level *P* < 0.05 except for paired *t-*test where statistical significance was defined as *P* < 0.01 to avoid spuriously significant results.

## Results

### Study population and baseline characteristics

Overall, 176 patients with cardiac AL amyloidosis were studied [106 male, 60%; mean age 64 (SD 11) years] (supplementary material online, *Figure S1*). All patients received first-line therapy with bortezomib. At baseline, the overall prevalence of diffuse subendocardial or transmural LGE was 162 of 176 (92%), with 66 (37%) of the subjects having transmural LGE and 96 (55%) having subendocardial LGE. Fourteen patients (8%) had diffusely elevated ECV but no discernible LGE. There was right ventricular LGE in 142 patients (81%).

### CMR findings at 6 months post-chemotherapy

A total of 142 patients had a CMR assessment 6 months after starting chemotherapy. Eighty-six (61%) patients achieved a good haematological response (59 patients, 42%, CR and 28 patients, 19%, VGPR). Twenty-seven percent of patients had PR and 12% had NR to chemotherapy.

**Figure 1:**
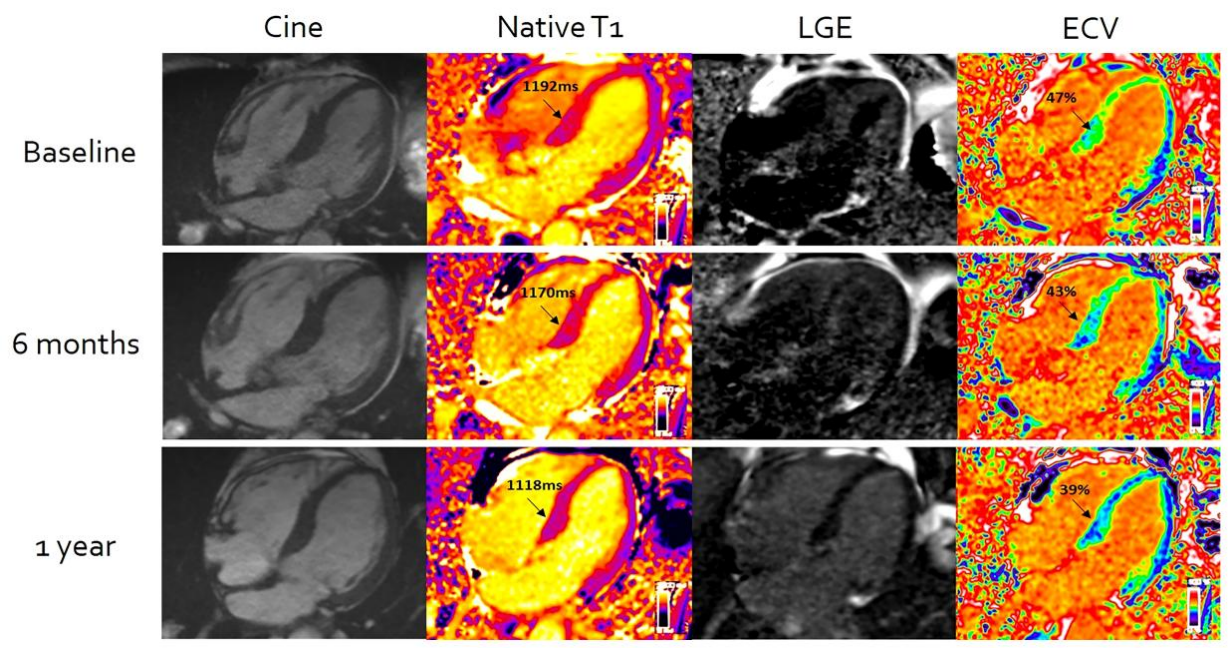
Cardiac systemic light-chain amyloid regression on serial cardiovascular magnetic resonance scans at baseline (top row) and after treatment with chemotherapy at 6 months (second row) and 1 year (third row). Reductions in native T1, late gadolinium enhancement, and extracellular volume within the myocardium are demonstrated progressively over the course of treatment.

**Figure 2:**
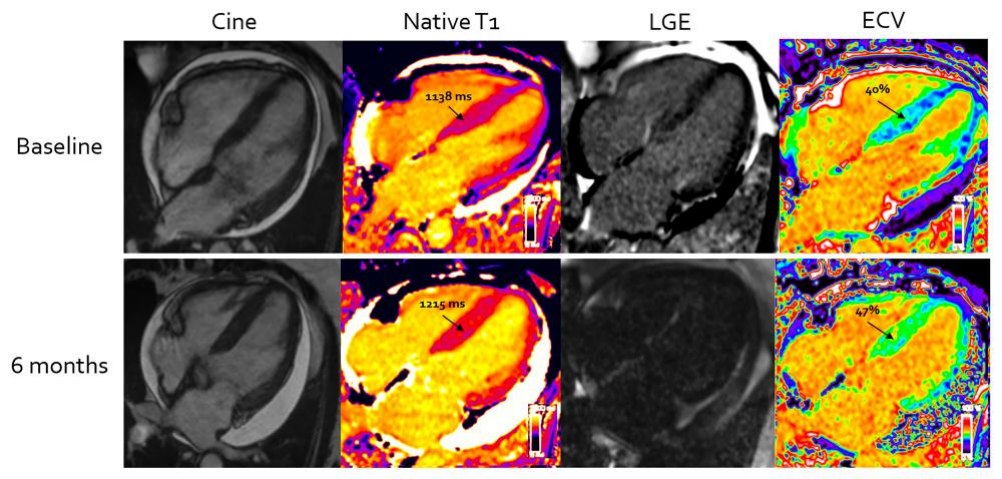
Cardiac systemic light-chain amyloid progression on serial cardiovascular magnetic resonance scans at baseline (top row) and after treatment with chemotherapy at 6 months (second row). Increase in native T1, late gadolinium enhancement, and extracellular volume within the myocardium are demonstrated after not achieving good response to chemotherapy at 6 months.

Reduction in ECV by CMR (classified by ≥5% decrease in ECV) (*Figure 1*) was detectable in 4 patients (3%) at 6 months after chemotherapy. Increased ECV by CMR (classified by ≥5% increase in ECV) (*Figure 2*) was detectable in 46 patients (32%) and 92 patients (65%) had stable ECV values (classified as <5% increase or decrease). Of the patients in CR, 4 patients had reduction in ECV, 45 patients had stable ECV values and 10 patients had increased ECV. Of the patients in VGPR, none showed reduction in ECV, 20 patients had stable ECV values and 8 patients had increased ECV.

Of the 46 (32%) patients with increased ECV by CMR, 61% were in a PR/NR and 39% in a VGPR/CR but their good haematological responses were not achieved until at least 3 months after chemotherapy had commenced. Of the 86 patients in CR/VGPR at 6 months, 43 patients (50%) achieved an early CR or VGPR (before 3 months of starting chemotherapy). Of those patients, 4 patients (9%) had reduction in ECV by CMR and 39 patients (91%) had stable findings. 43 patients (50%) achieved a late response (after 3 months of starting chemotherapy). Of those patients, 25 (58%) had stable findings and 18 (42%) had increased ECV by CMR.

All patients with reduction in ECV by CMR had a good haematological response (CR or VGPR), i.e. none of these patients had either PR or NR. Of the 92 (65%) patients with stable ECV findings, 65 (71%) were in a VGPR/CR and 27 (29%) in a PR/NR.

There were no significant differences in NT-proBNP, functional and structural parameters on the echocardiograms in patients who regressed. Among patients whose ECV increased, there was increasing of LV end-diastolic volume (LVEDV) [baseline 116 (SD 26) mL vs. 6 months 121 (SD 29) mL; *P* = 0.003], native T1 [baseline 1152 (SD 51) ms vs. 6 months 1191 (SD 67) ms; *P* = 0.001] and NT-proBNP [baseline median 1575 (IQR 816–5585) ng/L vs. 6 months 3594 (IQR 1483–11 004) ng/L] (*P* = 0.002).

Patients who had reduction in ECV by CMR at 6 months were younger and also had lower NT-proBNP levels, lower LV mass index and LV wall thickness, better longitudinal strain and smaller left atria at baseline (*Table 1*).

At 6 months, a total of 115 patients met the criteria to be classified as cardiac responders/non-responders by NT-proBNP measurements (response and progression of NT-proBNP were defined as changes that were both >30% and >300 ng/L, respectively; a baseline NT-proBNP ≥650 ng/L was required for NT-proBNP response to be evaluable).

There was cardiac response by NT-proBNP criteria in 37 patients (26%). Two of these patients (5%) had also reduction in ECV on CMR, 30 patients (81%) showed no significant changes in ECV and 5 (14%) had increased ECV. Seventy-eight patients had no cardiac response measured by NT-proBNP. Of these patients, 2 (3%) had reduction in ECV on CMR, 46 (59%) had stable findings and 30 (38%) had increased ECV.

### CMR findings at 1-year post-chemotherapy

A total of 121 patients had a CMR assessment 1 year after commencing chemotherapy that was associated with CR in 50 (41%) patients, VGPR in 35 (29%) patients, PR in 26 (22%) patients, and NR in 10 (8%).

Of the patients in CR, 20 patients had reduction in ECV, 24 patients had stable ECV measurements and 6 patients had increased ECV. Of the patients in VGPR, 7 patients had reduction in ECV, 24 patients had stable findings and 4 patients had increased ECV.

Reduction in ECV by CMR was evident in 27 patients (22%), all of whom had a CR or VGPR, whilst 67 patients (56%) had stable ECV findings and stable LGE pattern. There was increased ECV in 27 patients (22%), 17 of whom had a PR or NR; the remaining 10 patients with increased ECV had a CR or VGPR but their good haematological responses were not achieved until at least 6 months after chemotherapy had commenced.

Twelve (44%) patients with reduction in ECV demonstrated changes in the pattern of LGE (*Figure 1*); in two, LGE changed from a transmural to a subendocardial distribution, and in five cases from a subendocardial pattern to absence of visible LGE. In the remaining five patients, the subendocardial LGE present at baseline visually improved.

In the patients with reduction in ECV there was significant improvement in NT-proBNP [baseline median 2144 (IQR 442–6396) ng/L vs. 1 year 718 (IQR 153–1861) ng/L; *P* < 0.001], E/E′ ratio [baseline 16 (SD 7) vs. 1 year 13 (SD 5); *P* = 0.002], LVEDVi [baseline 62 (SD 14) mL/m^2^ vs. 1 year 67 (SD 13) mL/m^2^; *P* < 0.001] and LVESVi [baseline 22 (SD 9) mL/m^2^ vs. 26 (SD 11) mL/m^2^; *P* < 0.001]. By contrast, among patients whose ECV increased, there was worsening of LVEF [baseline 64% (SD 9) vs. 1 year 60% (SD 11); *P* = 0.009], native T1 [baseline 1144 (SD 56) ms vs. 1 year 1180 (SD 72) ms; *P* = 0.005] and NT-proBNP [baseline median 1575 (IQR 816–5585) ng/L vs. 1 year 3359 (IQR 1505–7563) ng/L; *P* = 0.002]. These findings are presented in *Table 2*.

**Table 2:** Biomarkers, echocardiographic and cardiovascular magnetic resonance findings in patients with AL according to extracellular volume changes by cardiovascular magnetic resonance at 1-year post-chemotherapy

	Regressors (*N* = 27)			Stable (*N* = 67)			Progressors (*N* = 27)		
	Baseline	1 year	*P*-value	Baseline	1 year	*P*-value	Baseline	1 year	*P*-value
Clonal response	CR = 20 (74%) VGPR = 7 (26%)			CR = 24 (36%) VGPR = 24 (36%) PR = 12 (18%) NR = 7 (10%)			CR = 6 (22%) VGPR = 4 (15%) PR = 14 (52%) NR = 3 (11%)		
**Biomarkers**									
NT-proBNP (ng/L)	2144 (442–6396)	718 (153–1861)	**<0.001**	2270 (812–4322)	1361 (465–3793)	**0.004**	1575 (816–5585)	3359 (1505–7563)	**0.002**
NT-proBNP response	Response = 15 (56%) No response = 3 (11%) 9 not classified (33%)			Response = 24 (36%) No response = 27 (40%) 16 not classified (24%)			Response = 3 (11%) No response = 18 (67%) 6 not classified (22%)		
6MWT (m)	447 (SD 92)	483 (SD 100)	0.035	420 (SD 113)	408 (SD 147)	0.626	401 (SD 146)	355 (SD 143)	0.246
**Echocardiographic parameters**									
IVS (cm)	1.4 (SD 0.2)	1.3 (SD 0.2)	0.129	1.5 (SD 0.2)	1.5 (SD 0.2)	0.341	1.4 (SD 0.3)	1.5 (SD 0.3)	0.09
LVEDD (cm)	40.1 (SD 6.9)	41.2 (SD 4.9)	0.639	41.3 (SD 5.8)	41.8 (SD 5.5)	0.166	42.0 (SD 4.8)	40.5 (SD 4.5)	0.052
LAA (cm^2^)	18.6 (SD 5.0)	16.5 (SD 3.4)	0.036	21.5 (SD 5.9)	21.2 (SD 5.8)	0.901	21.5 (SD 5.3	24.1 (SD 4.8)	**0.002**
E/E′	16 (SD 7)	13 (SD 5)	**0.002**	16 (SD 7)	15 (SD 6)	0.239	16 (SD 6)	17 (SD 6)	0.477
2D LS	-13 (SD 9)	-16 (SD 5)	0.040	-14 (SD 7)	-13 (SD 7)	0.855	-14 (SD 5)	-13 (SD 4)	0.050
**CMR parameters**									
LVEDVi (mL/m^2^)	62 (SD 14)	67 (SD 13)	**<0.001**	69 (SD 17)	73 (SD 17)	**0.004**	65 (SD 13)	70 (SD 13)	0.029
LVESVi (mL/m^2^)	22 (SD 9)	26 (SD 11)	**<0.001**	24 (SD 10)	27 (SD 12)	**0.007**	23 (SD 9)	28 (SD 10)	**0.006**
LV mass index (g/m^2^)	85 (SD 32)	82 ( SD 31)	0.268	101 (SD 29)	102 (SD 33)	0.700	97 (SD 32)	111 (SD 58)	0.088
LVSVi (mL/m^2^)	41 (SD 11)	44 (SD 7)	0.079	44 (SD 10)	46 (SD 10)	0.088	41 (SD 8)	42 (SD 9)	0.494
LVEF (%)	65 (SD 10)	64 (SD 10)	0.284	66 (SD 9)	64 (SD 10)	0.134	64 (SD 9)	60 (SD 11)	**0.009**
LAA (cm^2^)	23 (SD 5)	22 (SD 3)	0.050	28 (SD 8)	27 (SD 7)	0.011	27 (SD 6)	27 (SD 5)	0.943
RA area (cm^2^)	20 (SD 5)	20 (SD 3)	0.440	24 (SD 7)	23 (SD 7)	0.626	24 (SD 7)	24 (SD 6)	0.310
MAPSE (mm)	8 (SD 3)	9 (SD 3)	0.194	8 (SD 3)	8 (SD 2)	0.753	9 (SD 3)	8 (SD 2)	0.029
TAPSE (mm)	15 (SD 5)	17 (SD 5)	0.159	16 (SD 5)	16 (SD 6)	0.456	16 (SD 5)	14 (SD 5)	0.023
Native T1 (ms)	1149 (SD 83)	1127 (SD 58)	0.169	1147 (SD 53)	1152 (SD 54)	0.236	1144 (SD 56)	1180 (SD 72)	**0.005**
T2 (ms)	54 (SD 3)	52 (SD 2)	0.061	52 (SD 3)	54 (SD 3)	**0.020**	53 (SD 2)	54 (SD 4)	0.129
Visual LGE improvement	12 (44%) Transmural to subendocardial = 2 Improved subendocardial = 5 Subendocardial to no LGE = 5								

6MWT, 6-minute walk test; AL, systemic light-chain; CMR, cardiovascular magnetic resonance; CR, complete (haematological) response; LS, longitudinal strain; IVS, interventricular septum; LAA, left atrial area; LGE, late gadolinium enhancement; LVEDD, left ventricular end diastolic diameter; LVEDVi left ventricular end-diastolic volume indexed to body surface area; LVESVi, left ventricular end-systolic volume indexed to body surface area; LVSVi, left ventricular stroke volume indexed to body surface area; LVEF, left ventricular ejection fraction; MAPSE, mitral annular plane systolic excursion; NR, no (haematological) response; NT-proBNP, N-terminal pro-B-type natriuretic peptide; PR, partial (haematological) response, RA, right atrium; TAPSE, tricuspid annular plane systolic excursion; VGPR, very good partial (haematological) response.

All continuous variables are presented as mean and standard deviation with NT-proBNP presented as median and interquartile range although its natural logarithm was used for the paired comparisons.

In bold *P* < 0.01.

Patients who had reduction in ECV at 1 year had only smaller left atria at baseline (supplementary material online, *Table S1*).

At 1 year, a total of 90 patients could be classified in cardiac responders/non-responders by NT-proBNP. Forty-two patients had cardiac response measured by NT-proBNP, showing 15 (36%) with reduction in ECV by CMR, 24 (57%) stable findings and 3 (7%) increased ECV by CMR. Forty-eight patients had no cardiac response measured by NT-proBNP, of these, 3 patients (6%) had reduction in ECV by CMR, 27 (56%) stable findings and 18 (38%) increased ECV by CMR.

### CMR findings at 2 years post-chemotherapy

A total of 108 patients had a CMR assessment 2 years after commencing chemotherapy by which time 55 (51%) patients had achieved a CR, 31 (29%) patients a VGPR, 18 (17%) a PR and 4 (3%) NR. Of the patients in CR, 30 patients had reduction in ECV, 24 patients had stable ECV measurements and 1 patient had increased ECV. Of the patients in VGPR, 11 patients had reduction in ECV, 18 patients had stable findings and 2 patients had increased ECV.

**Figure 3:**
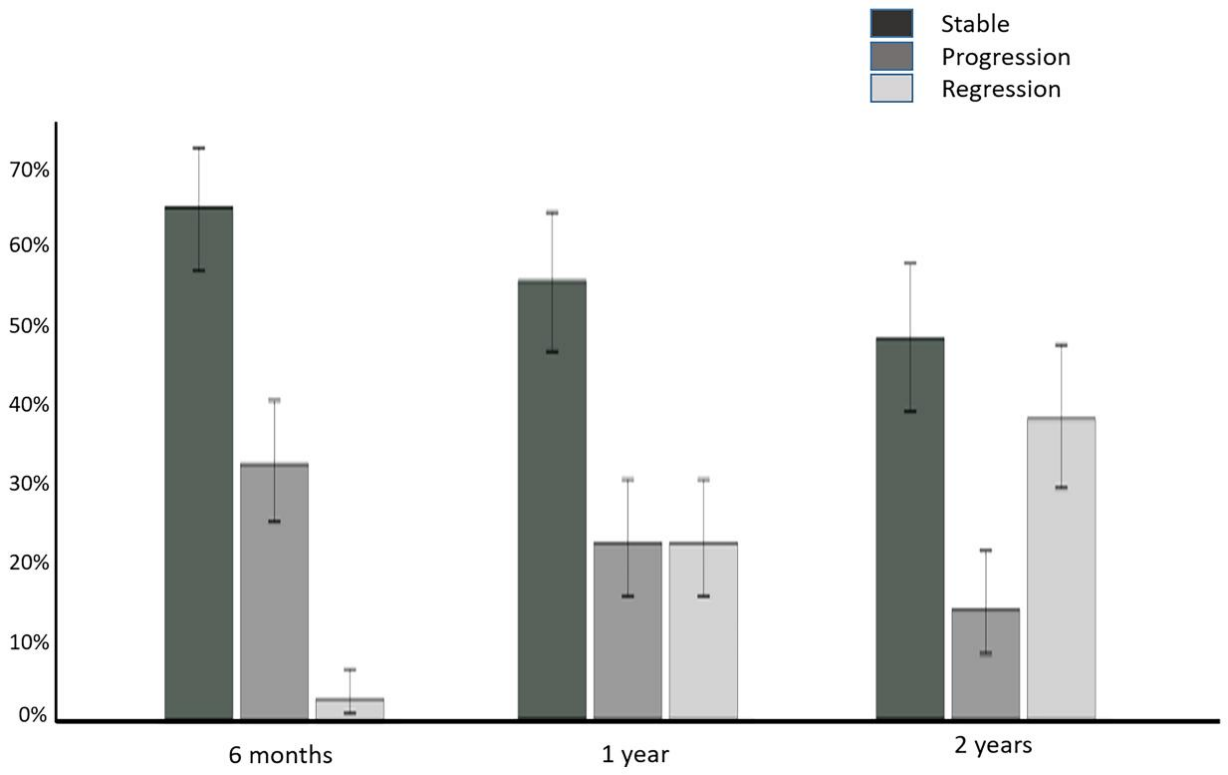
Bar chart with 95% confidence intervals, representing the proportion of patients with each grade of cardiovascular magnetic resonance response to chemotherapy at each time points studied (6 months, 1 year, and 2 years). Amyloid progression defined as ≥5% increase in extracellular volume, stable amyloid load defined as <5% change in extracellular volume and amyloid regression defined as ≥5% decrease in extracellular volume.

**Figure 4:**
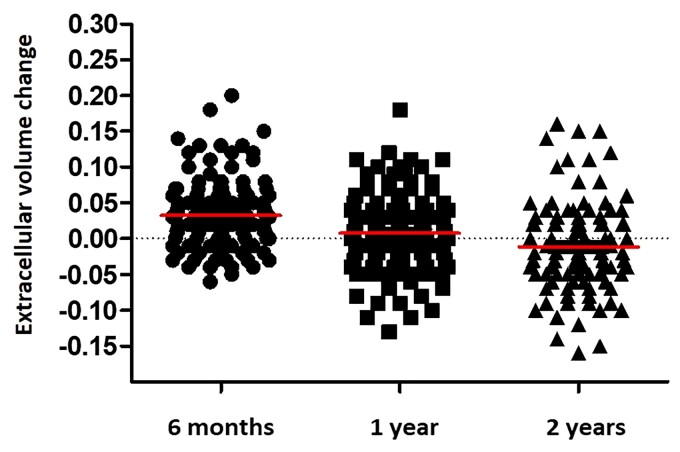
Dot plots comparing changes of extracellular volume from baseline to 6 months, 1 year after chemotherapy (solid horizontal lines represent group medians).

In total, reduction in ECV by CMR was present in 41 patients (38%) whilst increased ECV was evident in 15 patients (14%); stable ECV measurements and LGE pattern was present in 52 patients (48%). *Figure 3* is a bar chart representing the proportion of patients with each category of CMR response across the different time points studied (6 months, 1 year and 2 years). *Figure 4* is a dot plot graph comparing changes of ECV from baseline to 6 months, 1 year and 2 years after chemotherapy.

All patients with reduction in ECV had either a CR or VGPR haematological response (*P* < 0.05 compared with either PR or NR). However, favourable haematological responses were not always associated with amyloid regression. Three patients with CR at 2 years showed increased ECV, but this was in association with delays in their haematological responses until 6 months had elapsed in 2 cases and 12 months in the third.

Twenty-two^22^ patients (54%) with reduction in ECV by CMR had visual changes in the pattern of LGE (*Figure 1*). In four cases, the LGE changed from a transmural pattern to a subendocardial distribution and in 8 cases from a subendocardial pattern to absence of visible LGE. In the remaining 10 patients, the subendocardial LGE present at baseline visually improved.

In patients with reduction in ECV there was significant improvement in NT-proBNP [baseline median 2370 (939–4318) ng/L vs. 2 years 654 (IQR 315–1246) ng/L; *P* < 0.001], LVEDVi [baseline 65 (SD 16) mL/m^2^ vs. 2 years 69 (SD 14) mL/m^2^; *P* = 0.011], MAPSE [baseline 8 (SD 2) mm vs. 2 years 9 (SD 2) mm; *P* = 0.007] and native T1 [baseline 1147 (SD 69) ms vs. 2 years 1100 (SD 53) ms; *P* < 0.001].

By contrast, among patients whose ECV increased, there was only reduction in MAPSE [baseline 9 (SD 2) mm vs. 2 years 7 (SD 2) mm; *P* = 0.007] and TAPSE [baseline 16 (SD 5) mm vs. 2 years 12 (SD 4) mm; *P* = 0.001]. There were no significant changes in NT-proBNP among the patients who had increased ECV. These findings are presented in *Table 3*.

**Table 3:** Biomarkers, echocardiographic and cardiovascular magnetic resonance findings in patients with systemic light-chainaccording to extracellular volume changes by cardiovascular magnetic resonance at 2 years post-chemotherapy

	Regressors (*N* = 41)			Stable (*N* = 52)			Progressors (*N* = 15)		
	Baseline	2 years	*P*-value	Baseline	2 years	*P*-value	Baseline	2 years	*P*-value
Clonal response	CR = 30 (73%) VGPR = 11 (27%)			CR = 24 (46%) VGPR = 18 (35%) PR = 8 (15%) NR = 2 (4%)			CR = 1 (7%) VGPR = 2 (13%) PR = 10 (67%) NR = 2 (13%)		
**Biomarkers**									
NT-proBNP (ng/L)	2370 (939–4318)	654 (315–1246)	**0 < 0.001**	2326 (472–4840)	1410 (420–3727)	0.018	913 (806–5526)	2245 (1424–3876)	0.148
NT-proBNP response	Response = 28 (68%) No response = 6 (15%) 7 not classified (17%)			Response = 16 (30%) No response = 18 (35%) 18 not classified (35%)			Response = 2 (13%) No response = 10 (67%) 3 not classified (20%)		
6MWT (m)	448 ± 118	479 ± 121	0.126	406 ± 97	420 ± 109	0.894	376 ± 180	415 ± 152	0.827
**Echocardiographic parameters**									
IVS (cm)	1.4 ± 0.3	1.4 ± 0.2	0.101	1.4 ± 0.3	1.4 ± 0.3	0.891	1.4 ± 0.3	1.5 ± 0.2	0.020
LVEDD (cm)	41.1 ± 6.5	42.4 ± 5.0	0.243	41.9 ± 5.5	42.5 ± 5.6	0.327	41.8 ± 5.3	40.9 ± 5.9	0.485
LAA (cm^2^)	20.6 ± 6.0	20.0 ± 5.3	0.091	21.0 ± 5.2	21.1 ± 5.3	0.457	21.8 ± 3.6	22.1 ± 5.0	0.712
E/E′	15 ± 6	13 ± 5	0.059	16 ± 7	15 ± 6	0.643	16 ± 7	16 ± 5	0.763
2D LS	−14 ± 6	−15 ± 4	0.035	−15 ± 5	−13 ± 9	0.109	−14 ± 4	−10 ± 8	0.076
**CMR parameters**									
LVEDVi (mL/m^2^)	65 (SD 16)	69 (SD 14)	**0.011**	68 (SD 16)	72 (SD 17)	0.028	64 (SD 12)	73 (SD 28)	0.106
LVESVi (mL/m^2^)	23 (SD 10)	25 (SD 10)	0.035	24 (SD 10)	26 (SD 13)	0.200	22 (SD 10)	27 (SD 16)	0.051
LV mass index (g/m^2^)	92 (SD 35)	86 (SD 33)	0.022	103 (SD 38)	102 (SD 38)	0.575	101 (30)	116 (SD 35)	0.303
LVSVi (mL/m^2^)	43 (SD 10)	45 (SD 8)	0.114	44 (SD 9)	47 (SD 9)	**0.017**	42 (SD 8)	46 (SD 16)	0.227
LVEF (%)	66 ± 9	67 ± 10	0.674	65 ± 8	65 ± 11	0.814	67 ± 10	63 ± 12	0.029
LAA (cm^2^)	26 ± 7	25 ± 6	**0.002**	27 ± 7	25 ± 5	**0.006**	27 ± 6	27 ± 5	0.616
RA area (cm^2^)	22 ± 6	21 ± 5	0.494	22 ± 5	22 ± 5	0.483	25 ± 8	26 ± 7	0.238
MAPSE (mm)	8 ± 2	9 ± 2	**0.007**	9 ± 4	8 ± 2	0.280	9 ± 2	7 ± 2	**0.007**
TAPSE (mm)	16 ± 5	18 ± 5	0.015	17 ± 5	16 ± 6	0.050	16 ± 5	12 ± 4	**0.001**
Native T1 (ms)	1147 ± 69	1100 ± 53	**0 < 0.001**	1139 ± 57	1119 ± 72	0.025	1132 ± 41	1155 ± 44	0.030
T2 (ms)	53 ± 3	52 ± 3	0.038	53 ± 3	53 ± 3	0.235	52 ± 2	52 ± 3	0.840
Visual LGE improvement	22 (54%) Transmural to subendocardial = 4 Improved subendocardial = 10 Subendocardial to no LGE = 8								

6MWT, 6-minute walk test; AL, systemic light-chain; CMR, cardiovascular magnetic resonance; CR, complete (haematological) response; LS, longitudinal strain; LAA, left atrial area; LGE, late gadolinium enhancement; LVEDD, left ventricular end-diastolic diameter; LVEDVi left ventricular end-diastolic volume indexed by body surface area; LVESVi, left ventricular end-systolic volume indexed by body surface area; LVSVi, left ventricular stroke volume indexed by body surface area; LVEF, left ventricular ejection fraction; MAPSE, mitral annular plane systolic excursion; NR, no (haematological) response; NT-proBNP, N-terminal pro-B-type natriuretic peptide; PR, partial (haematological) response, RA, right atrium; TAPSE, tricuspid annular plane systolic excursion; VGPR, very good partial (haematological) response.

All continuous variables are presented as mean and standard deviation with NT-proBNP presented as median and interquartile range although its natural logarithm was used for the paired comparisons.

In bold *P* < 0.01.

There were no differences in any baseline characteristic amongst patients who had reduction in ECV by CMR at 2 years (supplementary material online, *Table S2*).

At 2 years, a total of 80 patients could be classified as cardiac responders/non-responders by NT-proBNP. Forty-six patients had cardiac response measured by NT-proBNP, of these, 28 patients (61%) had reduction in ECV by CMR, 16 (35%) stable findings and 2 (4%) increased ECV. 34 patients had no cardiac response by NT-proBNP, of these 6 patients (18%) had reduction in ECV, 18 (53%) stable findings and 10 (29%) increased ECV.

Thirty-nine patients had sustained CR for all the duration of the follow-up. Twenty-six patients (67%) had reduction on ECV by CMR at 2 years and 13 patients (33%) were stable at 2 years (no significant changes in ECV).

### Survival from baseline

**Figure 5:**
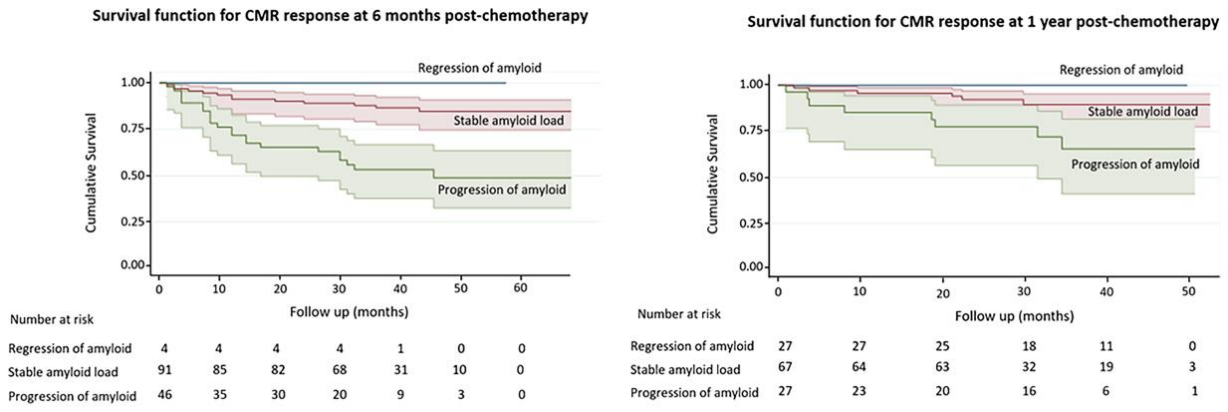
Kaplan–Meier survival curves, with shaded 95% confidence regions, displaying survival in all patients according to change in amyloid burden (measured by the change in extracellular volume on follow-up cardiovascular magnetic resonance) after 6 months (left panel) and 1 year of chemotherapy (right panel).

At follow-up, mean 40 ± 15 months, 36 (25%) of the 142 patients who had a CMR 6 months after commencing chemotherapy had died whereas no patients with reduction in ECV died during the entire follow-up period. *Figure 5* shows Kaplan-Meier survival curves according to changes in ECV by CMR at 6 months and 1 year.

In patients with stable ECV measurements at 6 months, survival probability at 24 months was 0.85 (95% CI 0.77–0.93) and at 36 months was 0.82 (95% CI 0.72–0.92). In patients with increased ECV at 6 months, survival probability at 24 months was 0.52 (95% CI 0.36–0.68) and at 36 months was 0.46 (95% CI 0.28–0.64).

In patients with stable ECV measurements at 1 year, survival probability at both 24 and 36 months was 0.90 (95% CI 0.82–0.98). In patients with increased ECV at 1 year, survival probability at both 24 and 36 months was 0.67 (95% CI 0.47–0.87).

Change in ECV at 6 months predicted death (progression HR 3.82; 95% CI 1.95–7.49; *P* < 0.001). Change in ECV at 6 months remained independently associated with prognosis after adjusting for haematological response, change in NT-proBNP and change in global longitudinal strain (progression HR 3.77; 95% CI 1.58–8.97; *P* < 0.01) (*Table 4*). Haematological response also remained independently associated with prognosis. Change in ECV at 1 year also predicted death (progression HR 4.463; 95% CI 1.753–11.359; *P* < 0.01).

**Table 4:** Univariate and multivariate analysis of mortality risk at 6 months after chemotherapy

		Univariate		Multivariate	
		HR (95% CI)	*P*-value	HR (95% CI)	*P*-value
CMR response	Stable	reference		reference	
	Progression	3.82 (1.95–7.49)	<0.001	3.77 (1.58–8.97)	0.003
LS response	Improvement	reference		reference	
	No improvement	1.36 (0.52–3.56)	0.526	0.95 (0.82–1.11)	0.544
NT-proBNP response	Improvement	reference		reference	
	Stable	1.54 (0.55–4.34)	0.410	0.98 (0.33–2.94)	0.971
	Worsening	2.71 (1.07–6.87)	0.036	1.22 (0.46–3.28)	0.689
Haematological response	Complete response	reference		reference	
	Very good partial response	2.11 (0.68–6.53)	0.197	2.18 (0.57–8.32)	0.257
	Partial response	4.16 (1.60–10.83)	0.004	4.39 (1.33–14.47)	0.015
	No response	9.03 (3.27–24.90)	<0.001	8.15 (2.10–31.56)	0.002

CI, confidence interval; CMR, cardiovascular magnetic resonance; LS, longitudinal strain; HR, hazard ratio; NT-proBNP, N-terminal pro-B-type natriuretic peptide.

In a subgroup analysis, changes in ECV discriminate groups with different survival amongst patients with CR/VGPR at 6 months (supplementary material online, *Figure S2*) and amongst the NT-proBNP no responders (supplementary material online, *Figure S3*). Changes in ECV were not predictor of survival amongst NT-proBNP responders, as the very small sample size implies lack of power to find a significant effect.

## Discussion

In this prospective study, we show that CMR with ECV measurements can track changes in patients with AL cardiac amyloid deposits over time, which most likely represent changes in the cardiac amyloid burden. ECV can track not just progression with unsuccessful chemotherapy, but also demonstrates reduction, which is most likely to represent amyloid regression when FLC precursors are removed by effective chemotherapy. The study shows that whilst deep haematological responses are required to attain reduction in ECV in keeping with cardiac amyloid regression, deep haematological response is not sufficient on its own, demonstrated here by patients with similar haematological responses being associated with quite different cardiac responses. Finally, changes in ECV independently correlate with prognosis after adjusting for known predictors, highlighting the role of CMR and T1 mapping refining treatment response in cardiac AL amyloidosis (*Structured Graphical Abstract*).

The progress in drug therapies developed for multiple myeloma has translated into improved outcomes in AL amyloidosis.^30^ Median survival in patients with AL amyloidosis has nearly doubled over the past decade^31^ but mortality in the first year after diagnosis remains high, reflecting the high incidence of advanced cardiac involvement. There are two interrelated measures of response to treatment in AL amyloidosis—the haematological response and cardiac response. The measurement of FLC has proven to be a robust marker of haematological response, VGPR and CR being associated with much improved survival.^32^ Cardiac organ response has historically been sought using NT-proBNP. However, NT-proBNP plasma concentration is influenced by many cardiac and non-cardiac factors, including kidney function, and often falls within just weeks of a favourable haematological response having been achieved. This very early decrease in NT-proBNP is thought to reflect diminished cardiotoxicity resulting from reduced abundance of harmful pre-fibrillar light chain aggregates as opposed to a reduction in myocardial amyloid burden. Whatever the mechanism, falls in NT-proBNP concentration of 30% or 300 ng/L from baseline following a clonal response to chemotherapy are associated with favourable clinical outcomes.^23^ The absence of significant echocardiographic improvements in a wide range of structural and functional markers under these circumstances, with the sole exception of longitudinal strain at 12 months in some patients,^4^ has propagated the belief that cardiac amyloid burden may only stabilize or improve minimally following successful chemotherapy. The emergence of advanced myocardial tissue characterization by magnetic resonance, specifically T1 mapping with ECV mapping has made possible to estimate cardiac amyloid load *in vivo*.

The present study has focused on the cardiac amyloid response, by measuring the changes in ECV at three different time points after the start of chemotherapy, 6 months, 12 months and 2 years. Contrast agent administration and ECV measurements by CMR enable the isolation of the signal from the extracellular space. Amyloidosis is the exemplary interstitial disease of the myocardium, where amyloid deposits in the extracellular space cause a significant increase in the ECV. Therefore, a global increase in ECV is most likely to represent cardiac amyloid burden. By 6 months since starting chemotherapy, very few patients have evidence of reduction in ECV of consistent with myocardial amyloid regression. However, one third have evidence of increased ECV in keeping with amyloid progression, of which 39% have achieved a good haematological response. The patients in CR/VGPR who had increased ECV in keeping with amyloid progression did not achieve good haematological response until at least 3 months after chemotherapy. This observation adds nuances to the hypothesis of the importance of achieving a deep reduction in the FLC (i.e. CR or VGPR) in order to attain a cardiac response—amyloid will continue to be deposited until a sufficient haematological response is obtained. This strongly supports the need for not only achieving a deep haematological response, but also achieve the response as rapidly as possible. At 1 year post-chemotherapy, 22% presented increased ECV in keeping with amyloid progression, but most encouragingly, a similar proportion of patients had reduction in changes in ECV consistent with amyloid regression, substantially more than at 6 months, having 44% of these patients also significant changes in the pattern of LGE. All patients with reduction in ECV in keeping with cardiac amyloid regression had achieved a CR or VGPR, confirming the importance of a deep reduction in the amyloid production. However, not all patients who experienced a deep and long-lasting haematological response had reduction in ECV, suggesting that this is not sufficient on its own. This is in keeping with the hypothesis, already proven in other organs, that amyloid deposition is a dynamic process, with a constant turnover of amyloid such that accumulation of amyloid occurs when the rate of formation exceeds the rate of clearance, and regression when it less.^33^ It is noteworthy that increased ECV in consistent with progression of amyloid was demonstrated at 1-year follow-up in some patients whose excellent haematological responses had taken more than 6 months to occur. At the 2-year stage, there had been reduction in ECV in keeping with regression in a very substantial 38% of cases, but all of these patter patients had achieved their favourable haematological responses within the first 6 months.

Echocardiography failed to demonstrate any differences in functional and structural parameters among patients who had reduction, increase (with the only exception of a small increase in LVEDV) or stable ECV at 6 months. However, by 1 year, reduction in ECV was associated with improvement in E/E′ and eccentric remodelling (higher EDV, ESV). At 2 years there was evidence of ongoing further remodelling with improvement in MAPSE. In patients with increased ECV, the pattern of functional and structural changes was of a predominant reduction in LVEF at 1 year and MAPSE at 2 years. Overall, these findings are in keeping with ECV likely being able to detect changes in myocardial composition at the earliest stage, as early as 6 months, with other markers of myocardial remodelling becoming evident later on.

Finally, our study also demonstrated that changes in ECV were able to predict death as early as 6 months into treatment even when adjusted for the haematological response, change in NT-proBNP and longitudinal strain highlighting the role of CMR in refining treatment response beyond the reduction in FLC and NT-proBNP by directly imaging the myocardial substrate.

These results could have important clinical implications for the management of patients with AL amyloidosis. The current data, which demonstrate changes in ECV (as a reflection of amyloid burden) are significantly associated with overall survival, are an important addition to the standard armamentarium of amyloid organ assessment. In a patient with serial tracking on ECV, if there is lack of ECV response and the patient has not achieved a complete haematological response, these data strongly raise the question whether such a patient will benefit from further treatment conversely in a patient with less than a haematological response and an ECV response would obviate this need. Hence, the current ECV change data is a new and novel addition to methods for amyloid assessment. These data can form the basis of future studies where formal assessment of treatment change using this method and its impact on outcomes needs to be undertaken. In summary, the future management of cardiac amyloidosis is likely to be a multidimensional approach, where haematological, NT-proBNP response and CMR response will have a different role at different time points and the combination of these markers will depict a comprehensive clinical picture that could help clinicians to better tailor chemotherapy treatment in each individual patient. Furthermore, there are also several compounds at different stages of development, that promote regression by directly targeting and enhancing the clearance of existing amyloid deposits.^34–36^ The ability to measure changes in cardiac amyloid load over time could be of great value as an endpoint in early-stage drug development and dose ranging.

This study has several limitations. There is a survival bias in that we quote only subjects with follow-up scans—it may be that the extent of differences is underestimated if, for example, PR or NR subjects for whom amyloid accumulates die before follow-up scanning. Patients who had contraindications for CMR after the baseline scan (due to renal impairment, pacemaker implantation, or difficulties to lie flat) were excluded. The first follow-up CMR was performed at 6 months, limiting the utility of this study for first-line treatment. Future studies should explore earlier time points, such as 3 months from the start of chemotherapy. Finally, some of the patients decided to be followed up locally and therefore there was a lost to follow-up in our centre (supplementary material online, Figure S1).

## Supplementary Material

ehac363_Supplementary_Data

## Data Availability

The data underlying this article are available in the article and in its online supplementary material.
