# Supplementary material for: Cardiovascular magnetic resonance in light-chain amyloidosis to guide treatment
Source: Eur Heart J. 2022 Jul 26;43(45):4722–35. doi: 10.1093/eurheartj/ehac363 (PMC9712028; doi:10.1093/eurheartj/ehac363)
Supplement: ehac363_Supplementary_Data [file ehac363_supplementary_data.zip › Supplementary table 1.docx]

**Supplementary table 1.** Baseline characteristics, biomarkers, 6MWT, echocardiographic and CMR parameters for patients who had amyloid regression, stable findings or progression by CMR at 1 year.

| **Characteristics** | **Regression**  27 patients | **Stable**  67 patients | **Progression**  27 patients |
| --- | --- | --- | --- |
| **Sex** | |  |  |
| Men, N (%) | 15 (56%) | 40 (60%) | 18 (67%) |
| Women, N (%) | 12 (44%) | 27 (40%) | 9 (33%) |
| **Age** (y) | 60 (14) | 65 (10) | 64 (12) |
| **Biomarkers** | | | |
| NT-proBNP (pmol/L) | 2144 (442-6396) | 2270 (812-4322) | 1575 (816-5585) |
| **6MWT** (m) | 447 (92) | 420 (113) | 401 (146) |
| **Echocardiographic parameters** | | | |
| IVS (cm) | 1.40 (SD 0.23) | 1.45 (SD 0.24) | 1.44 (SD 0.27) |
| LPW (cm) | 1.40 (SD 0.24) | 1.46 (SD 0.24) | 1.42 (SD 0.23) |
| LVEDD (cm) | 4.10 (SD 0.69) | 4.13 (SD 0.58) | 4.20 (SD 0.48) |
| LAA (cm^2^) | 18.57 (SD 5.05) | 21.53 (SD 5.89) | 21.50 (SD 5.27) |
| Average E’ (cm/s) | 0.07 (SD 0.03) | 0.07 (SD 0.03) | 0.08 (SD 0.08) |
| E/E’ | 16 (SD 7) | 16 (SD 7) | 16 (SD 6) |
| E wave DT (msec) | 171 (SD 64) | 162 (SD 54) | 181 (SD 51) |
| 2D LS | -14.4 (SD 5.6) | -14.2 (SD 5.2) | -13.79 (SD 4.5) |
| **CMR parameters** | | | |
| LVEDV_i_ (mL/m^2^) | 62 (SD 14) | 69 (SD 17) | 65 (SD 13) |
| LVESV_i_ (mL/m^2^) | 22 (SD 9) | 24 (SD 10) | 23 (SD 9) |
| Maximal IVS (mm) | 14 (SD 3) | 16 (SD 4) | 17 (SD 5) |
| LV mass_i_ (g/m^2^) | 85 (SD 32) | 101 (SD 29) | 97 (SD 32) |
| LVSV_i_ (mL/m^2^) | 41 (SD 11) | 44 (SD 10) | 41 (SD 8) |
| LVEF (%) | 65 (SD 10) | 65 (SD 9) | 64 (SD 9) |
| LAA (cm^2^) | 23 (SD 5) | 28 (SD 8)* | 27 (SD 6) |
| TAPSE (mm) | 15 (SD 5) | 16 (SD 6) | 16 (SD 5) |
| Native T1 (msec) | 1149 (SD 83) | 1147 (SD 53) | 1144 (SD 56) |
| T2 (msec) | 53 (SD 4) | 52 (SD 3) | 53 (SD 2) |
| ECV (%) | 47 (SD 8) | 46 (SD 7) | 45 (SD 7) |

6MWT, 6-minute walk test; AL, light-chain amyloidosis; CMR, cardiovascular magnetic resonance; CR: complete (haematological) response, DT, deceleration time; ECV, extracellular volume; LS, longitudinal strain; IVS, interventricular septum; LAA, left atrial area; LPW, left posterior wall; LV, left ventricle; LVEDD, left ventricular end diastolic diameter; LVEDVi, left ventricular end diastolic volume indexed by body surface area; LVEF, left ventricular ejection fraction; LVESVi, left ventricular end systolic volume indexed by body surface area; LVSVi, left ventricular stroke volume indexed by body surface area; LVEF, left ventricular ejection fraction; NR, no (haematological) response; NT-proBNP, N-terminal pro-brain natriuretic peptide; PR, partial (haematological) response; TAPSE, tricuspid annular plane systolic excursion; VGPR, very good partial (haematological) response.

All continuous variables are presented as mean and standard deviation apart from NT-proBNP which is presented as median and interquartile range. Natural log-transformed NT-proBNP was used for parametric testing, but in this table the raw data is summarized by the median and interquartile range.

P-values for pairwise comparison: *= P<0.05 for regression vs stable, **=P<0.05 for regression vs progression.
