# Supplementary material for: Cardiovascular magnetic resonance in light-chain amyloidosis to guide treatment
Source: Eur Heart J. 2022 Jul 26;43(45):4722–35. doi: 10.1093/eurheartj/ehac363 (PMC9712028; doi:10.1093/eurheartj/ehac363)
Supplement: ehac363_Supplementary_Data [file ehac363_supplementary_data.zip › Supplementary table 2.docx]

**Supplementary table 2.** Baseline characteristics, biomarkers, 6MWT, echocardiographic and CMR parameters for patients who had amyloid regression, stable findings or progression by CMR at 2 years.

| **Characteristics** | **Regression**  41 patients | **Stable**  52 patients | **Progression**  15 patients |
| --- | --- | --- | --- |
| **Sex** | |  |  |
| Men, N (%) | 27 (66%) | 30 (58%) | 11 (73%) |
| Women, N (%) | 14 (34%) | 22 (42%) | 4 (27%) |
| **Age** (y) | 63 (11) | 66 (9) | 60 (12) |
| **Biomarkers** | | | |
| NT-proBNP (pmol/L) | 2369 (938-4317) | 2327 (471-4840) | 913 (806-5526) |
| **6MWT** (m) | 447 (118) | 406 (97) | 376 (180) |
| **Echocardiographic parameters** | | | |
| IVS (cm) | 1.43 (SD 0.25) | 1.42 (SD 0.27) | 1.40 (SD 0.25) |
| LPW (cm) | 1.41 (SD 0.24) | 1.41 (SD 0.25) | 1.43 (SD 0.23) |
| LVEDD (cm) | 4.11 (SD 0.65) | 4.19 (SD 0.55) | 4.18 (SD 0.53) |
| LAA (cm^2^) | 20.54 (SD 6.01) | 20.93 (SD 5.20) | 21.82 (SD 3.50) |
| Average E’ (cm/s) | 0.07 (SD 0.03) | 0.07 (SD 0.03) | 0.09 (SD 0.09) |
| E/E’ | 15 (SD 6) | 16 (SD 6) | 16 (SD 8) |
| E wave DT (msec) | 161 (SD 56) | 178 (SD 53) | 173 (SD 61) |
| 2D LS | -14.2 (SD 4.5) | -14.8 (SD 5.1) | -13.68 (SD 3.6) |
| **CMR parameters** | | | |
| LVEDV_i_ (mL/m^2^) | 65 (SD 16) | 68 (SD 16) | 64 (SD 12) |
| LVESV_i_ (mL/m^2^) | 23 (SD 10) | 24 (SD 10) | 22 (SD 9) |
| Maximal IVS (mm) | 15 (SD 4) | 16 (SD 5) | 17 (SD 4) |
| LV mass_i_ (g/m^2^) | 92 (SD 35) | 103 (SD 38) | 101 (SD 30) |
| LVSV_i_ (mL/m^2^) | 43 (SD 10) | 44 (SD 9) | 42 (SD 8) |
| LVEF (%) | 66 (SD 9) | 65 (SD 8) | 67 (SD 10) |
| LAA (cm^2^) | 26 (SD 7) | 27 (SD 7) | 27 (SD 6) |
| TAPSE (mm) | 16 (SD 5) | 17 (SD 5) | 16 (SD 5) |
| Native T1 (msec) | 1147 (SD 69) | 1139 (SD 57) | 1132 (SD 41) |
| T2 (msec) | 53 (SD 3) | 53 (SD 3) | 52 (SD 2) |
| ECV (%) | 46 (SD 8) | 44 (SD 7) | 44 (SD 5) |

6MWT, 6-minute walk test; AL, light-chain amyloidosis; CMR, cardiovascular magnetic resonance; CR: complete (haematological) response, DT, deceleration time; ECV, extracellular volume; LS, longitudinal strain; IVS, interventricular septum; LAA, left atrial area; LPW, left posterior wall; LV, left ventricle; LVEDD, left ventricular end diastolic diameter; LVEDVi, left ventricular end diastolic volume indexed by body surface area; LVEF, left ventricular ejection fraction; LVESVi, left ventricular end systolic volume indexed by body surface area; LVSVi, left ventricular stroke volume indexed by body surface area; LVEF, left ventricular ejection fraction; NR, no (haematological) response; NT-proBNP, N-terminal pro-brain natriuretic peptide; PR, partial (haematological) response; TAPSE, tricuspid annular plane systolic excursion; VGPR, very good partial (haematological) response.

All continuous variables are presented as mean and standard deviation apart from NT-proBNP which is presented as median and interquartile range. Natural log-transformed NT-proBNP was used for parametric testing, but in this table the raw data is summarized by the median and interquartile range.
