# Supplementary figures and images for: Cardiovascular magnetic resonance in light-chain amyloidosis to guide treatment
Source: Eur Heart J. 2022 Jul 26;43(45):4722–35. doi: 10.1093/eurheartj/ehac363 (PMC9712028; doi:10.1093/eurheartj/ehac363)

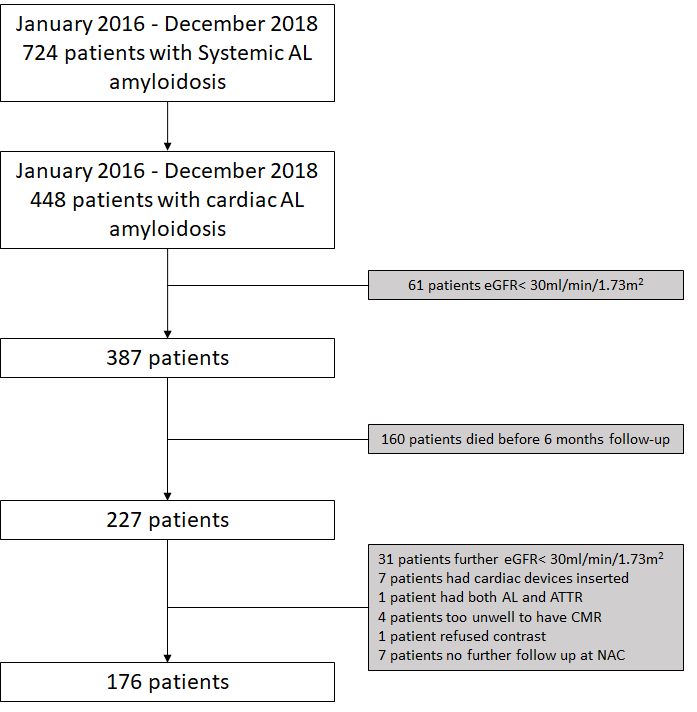

Supplement: ehac363_Supplementary_Data [file ehac363_supplementary_data.zip › Supplementary figure 1.tif]

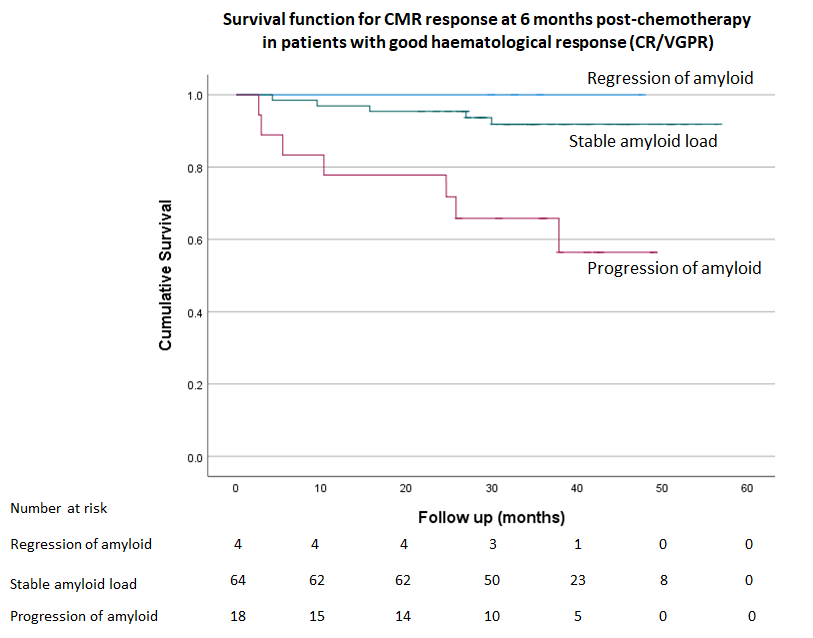

Supplement: ehac363_Supplementary_Data [file ehac363_supplementary_data.zip › Supplementary figure 2.tif]

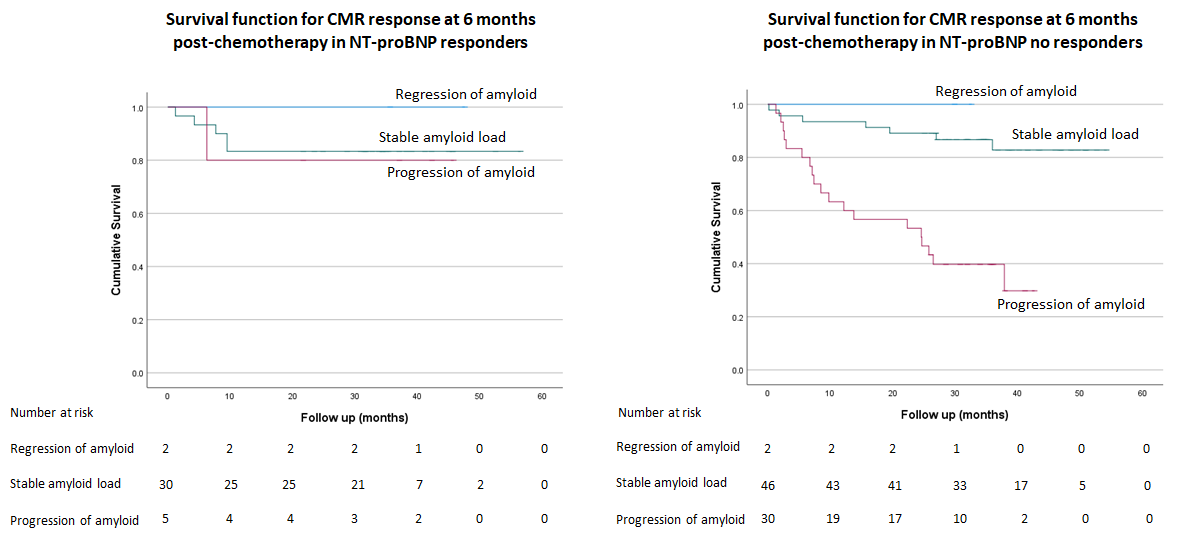

Supplement: ehac363_Supplementary_Data [file ehac363_supplementary_data.zip › Supplementary figure 3.tif]
